# Ultrasound-based deep learning using the VGGNet model for the differentiation of benign and malignant thyroid nodules: A meta-analysis

**DOI:** 10.3389/fonc.2022.944859

**Published:** 2022-09-28

**Authors:** Pei-Shan Zhu, Yu-Rui Zhang, Jia-Yu Ren, Qiao-Li Li, Ming Chen, Tian Sang, Wen-Xiao Li, Jun Li, Xin-Wu Cui

**Affiliations:** ^1^ Department of Ultrasound, the First Affiliated Hospital of Medical College, Shihezi University, Shihezi, China; ^2^ Department of Medical Ultrasound, Tongji Hospital, Tongji Medical College, Huazhong University of Science and Technology, Wuhan, China; ^3^ NHC Key Laboratory of Prevention and Treatment of Central Asia High Incidence Diseases, First Affiliated Hospital, School of Medicine, Shihezi University, Shihezi, China

**Keywords:** meta-analysis, ultrasound, thyroid nodules, deep learning, VGGNet

## Abstract

**Objective:**

The aim of this study was to evaluate the accuracy of deep learning using the convolutional neural network VGGNet model in distinguishing benign and malignant thyroid nodules based on ultrasound images.

**Methods:**

Relevant studies were selected from PubMed, Embase, Cochrane Library, China National Knowledge Infrastructure (CNKI), and Wanfang databases, which used the deep learning-related convolutional neural network VGGNet model to classify benign and malignant thyroid nodules based on ultrasound images. Cytology and pathology were used as gold standards. Furthermore, reported eligibility and risk bias were assessed using the QUADAS-2 tool, and the diagnostic accuracy of deep learning VGGNet was analyzed with pooled sensitivity, pooled specificity, diagnostic odds ratio, and the area under the curve.

**Results:**

A total of 11 studies were included in this meta-analysis. The overall estimates of sensitivity and specificity were 0.87 [95% CI (0.83, 0.91)] and 0.85 [95% CI (0.79, 0.90)], respectively. The diagnostic odds ratio was 38.79 [95% CI (22.49, 66.91)]. The area under the curve was 0.93 [95% CI (0.90, 0.95)]. No obvious publication bias was found.

**Conclusion:**

Deep learning using the convolutional neural network VGGNet model based on ultrasound images performed good diagnostic efficacy in distinguishing benign and malignant thyroid nodules.

**Systematic Review Registration:**

https://www.crd.york.ac.nk/prospero, identifier CRD42022336701.

## Introduction

Thyroid nodules are the most common diseases of the endocrine system, with an ultrasound population detection rate of about 65%, of which approximately 10% is thyroid cancer (1). Thyroid cancer, despite the low incidence, is one of the fastest growing of all cancer types, having increased approximately 2.4 times in the last 30 years (2). It has become a public health concern in most parts of the world. Therefore, early detection and early accurate diagnosis of benign and malignant thyroid nodules are crucial to develop treatment plans and predict prognosis for patients with thyroid nodules, yet this is a great challenge for radiologists and physicians.

Ultrasound is currently the first-line examination of choice for the clinical diagnosis of thyroid nodules, and it is not only the main method for cancer risk stratification of thyroid nodules, but also usually used for the guidance of fine-needle aspiration biopsy. However, the differential diagnosis of thyroid nodules by 2D ultrasound has certain limitations. The quality of ultrasound images is susceptible to many factors, such as the cooperation of patients, the performance of ultrasound machines, and the operating techniques of radiologists (3). In addition, ultrasound diagnostic results are affected by the experience level of radiologists, and the recognition of ultrasound image characteristics of nodules differs among radiologists with different working experience, which is subjective (4). Therefore, there is an urgent need to explore a diagnostic tool that is noninvasive, accurate, and objective in the differential diagnosis of the benign and malignant thyroid nodules preoperatively.

In 2013, deep learning of artificial intelligence (AI) was ranked as one of the top 10 breakthrough technologies by *MIT Technology Review*, ranking no. 1. From then on, deep learning entered an era of rapid development and played a pivotal role in the medical field, especially in medical image recognition. Some studies used the deep learning convolutional neural network to extract ultrasound features to identify and diagnose benign and malignant thyroid nodules, and some of the studies with diagnostic performance could be comparable to or better than the advanced physicians, which could reduce unnecessary punctures and overtreatment, and help grassroots and inexperienced physicians improve diagnostic efficiency and accuracy (5–7). In addition, Lee et al. (8) explored the use of deep learning convolutional neural networks in predicting the presence of lymph node metastasis in thyroid cancer on ultrasound, and their results indicated good predictive diagnostic accuracy (accuracy of 83.0%). Accordingly, ultrasound-based AI provides a new direction and method for radiologists to accurately and non-invasively identify and diagnose benign and malignant thyroid nodules and predict lymph node metastasis in the neck before surgery.

Previous published AI studies on thyroid disease can be broadly classified into two categories: traditional machine learning (ML) and deep learning (DL). Traditional ML uses manual extraction of image features, but ultrasound images are highly variable and feature extraction is dependent on physician experience; therefore, the accuracy of diagnosing benign and malignant thyroid nodules varies between empirical practitioners. Deep learning is a development of machine learning using automated extraction of image features, which is independent of physician experience (9). Among them, convolutional neural network (CNN) is a well-known deep learning structure in the field of medical image analysis and is a fully trainable deep learning algorithm consisting of an input layer, a hidden layer, and an output layer (10, 11). The hidden layer usually contains a convolutional layer, a pooling layer, and a fully connected layer. Compared with traditional machine learning methods, CNN performs better in target detection and image classification, and can better extract semantic features (12). Nowadays, CNN is considered one of the most advanced methods among many representative algorithms of deep learning, and VGGNet is a widely used model in CNN algorithms (10, 11). This model is the first network structure to reach “deep” in a real sense, as it takes a different research direction from previous CNN models, namely, deepening the network, and proves that the deep network with small filters is superior to the shallow network with large filters (13). Therefore, the deep learning VGGNet model alone was selected as the research subject to avoid selection bias and ensure the stability and reliability of the results.

At present, a number of studies have demonstrated that using the deep learning VGGNet model can differentiate benign and malignant thyroid nodules on ultrasound to assist physicians in making diagnostic results, but the sensitivity of different studies varies. The sensitivity was 93% in the study results of Zhu et al. (5), but only 77% in the study results of Zhou et al. (14). The sensitivity of ultrasound-based deep learning VGGNet in the diagnosis of thyroid nodules was quite different, and no meta-analysis of ultrasound-based deep learning VGGNet models for the determination of the nature of thyroid nodules has been found. Therefore, this meta-analysis aims to evaluate the efficacy of the ultrasound-based deep learning VGGNet model in distinguishing and diagnosing the nature of thyroid nodules to help radiologists make more accurate diagnoses.

## Materials and methods

### Search strategy

This meta-analysis was a study summarizing previously published literature on the differential diagnosis of thyroid nodules with an ultrasound-based deep learning convolutional neural network VGGNet model, thus requiring no ethical confirmation or patient consent. The literature was independently searched in PubMed, Embase, Cochrane Libraries, China National Knowledge Infrastructure (CNKI), and Wanfang databases up to September 2021, updated as of June 2022. The main following keywords were searched: “Deep learning” or “DL” or “Neural network” and “ultrasonography” OR “ultrasound” OR “ultrasonic” or “diagnostic imaging” and “thyroid” or “thyroid gland” or “thyroid nodules”. Moreover, references of retrieved topic-related systematic reviews were also manually searched, and other relevant studies were read and identified to make the search more comprehensive.

### Study selection

Inclusion criteria were as follows (1): studies that used the deep learning VGGNet model for the differential diagnosis of benign and malignant thyroid nodules (2); at least one ultrasound imaging modality (3); literature that can provide true positives (TP), false positives (FP), false negatives (FN), and true negatives (TN) (4); test set data or validation set data would be chosen; if both were present at the same time, we chose to use the test set; if there were both external and internal test sets, we also conducted a meta-analysis on the external test sets; if there were more than one external test set results in a paper at the same time, we would remove the highest and lowest diagnostic performance results and select the intermediate results; and (5) the gold standard was fine-needle aspiration (FNA), pathology, or both.

Excluded studies include (1) studies that did not match the gold standard (2); convolutional neural network models unrelated to the deep learning VGGNet model (3); studies that did not provide the necessary 2×2 contingency data (4); literature with only abstracts, reviews, conference report, papers not published in journals, full text that were not accessible online, and so on; and (5) duplicate studies.

### Quality assessment and data extraction

The Quality Assessment of Diagnostic Accuracy Studies (QUADAS) tool is a recognized tool for quality assessment of diagnostic accuracy tests, because of its specific problem definition and clinical actionability that is widely used in diagnostic meta-analyses (15, 16). The QUADAS tool was revised in 2011 and was called QUADAS-2, consisting of four main parts: case selection, index test, reference standard, and flow and timing, and all components are evaluated in terms of risk of bias (17). The 11 studies included were independently evaluated by two reviewers using the QUADAS-2 tool, and resolved by discussion between internal members if a disagreement was encountered during the assessment. QUADAS-2 results were output using RevMan 5.3, the dedicated software for the Cochrane Collaboration Network.

In this study, two authors independently read the titles and abstracts to screen eligible papers, and then read the full text to determine the included papers. The information obtained from each study was extracted independently, including first author, year of publication, country, gold standard, training set size, test set size, fourfold table data (TP, FP, FN, and TN), sensitivity, specificity, VGGNet type, and testing objects. If fourfold table data were not available in the literature, they were excluded.

### Statistical analysis

The entire data from the included studies were selected using Excel 2019, and sensitivity, specificity, and diagnostic odds ratio (DOR) [95% confidence interval (CI)] were summarized using STATA software version 16.0. The area under the receiver operating characteristic (ROC) curve and 95% CI were also calculated, and the value of the diagnostic test was assessed by the area under the curve (AUC) value, where AUC < 0.70 means low diagnostic accuracy, 0.70 < AUC < 0.90 indicates moderate diagnostic accuracy, and AUC ≥ 0.90 indicates high diagnostic accuracy. Statistical inconsistency between studies was assessed using the *I* ^2^ and Cochrane *Q* tests; if *I* ^2^ < 50%, it will choose a fixed-effects model to assess sensitivity and specificity, and if *I* ^2^ > 50%, a random-effects model would be used. Meta-regression analysis was used and reasons were given when statistical heterogeneity was large. *p* < 0.05 was considered statistically significant.

## Results

### Literature searches

**Figure 1:**
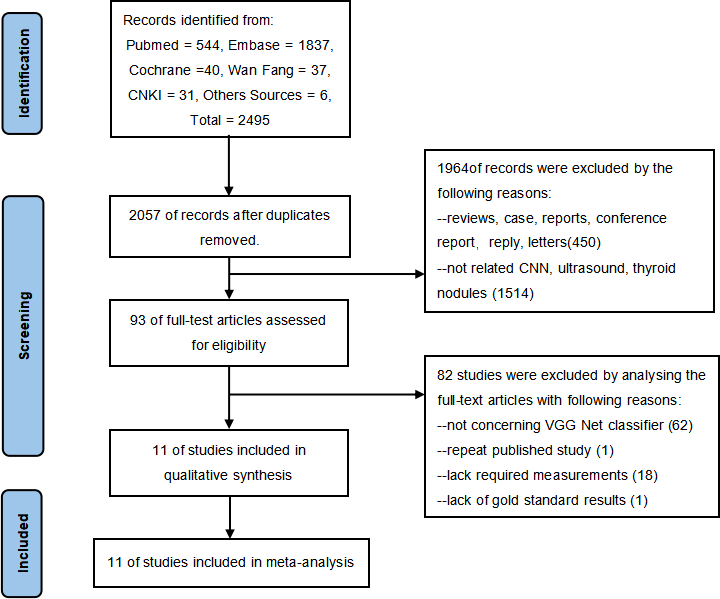
Study flowchart. Eleven studies were included in this meta-analysis.

Through a comprehensive search, 2,495 records were obtained for our study as of September 2021, updated as of June 2022, including 544 papers from PubMed, 1,837 papers from Embase, 40 articles from Cochrane Libraries, 31 papers from CNKI, 37 papers from Wanfang database, and 6 papers from other sources. After preliminarily eliminating duplicate literatures, two researchers independently read the titles and abstracts of the remaining literatures, excluding literature reviews, cases, news, and other research types. The full text of the literature obtained will be further read through and eventually include 11 studies eligible for the meta-analysis. The detailed selection procedure is shown in **Figure 1**.

### Study characteristics

We registered this meta-analysis on the PROSPERO website; the registration number is CRD42022336701. Following the PRISMA-Diagnostic studies selection process, we eventually included 11 papers; all studies are included in **Table 1**. The following are some basic characteristics of the included literature. All studies were published within the last 5 years. Eight papers used the deep learning VGG-16 model (14, 18–24). Four papers clearly indicated retrospective study (5, 6, 14, 19). Two papers did not give an explicit number of training sets (19, 20). Three papers compared the deep learning CNN algorithm with radiologists, and the results were comparable to or better than those of the advanced radiologists (5, 7, 23). Qin et al. (21) extracted both ultrasound image features and ultrasound elastic image features. Zhu et al. (5) only included thyroid nodules in female patients.

**Table 1:** Characteristics of the included studies.

Author	Year	Country	Gold standard	Training database		Test database		Se (%)	Sp (%)	TP	FP	FN	TN	VGG	Testing objects
				B	M	B	M								
Kwon S.W et al. (18)	2020	Korea	FNA / pathology	199	260	62	83	0.92	0.70	76	19	7	43	16	Interior
Liu Z et al. (19)	2021	China	FNA	–	–	67	96	0.79	0.87	76	9	20	58	16	Interior
Wu K et al. (20)	2020	China	pathology	–	–	520	636	0.86	0.78	547	114	89	406	16	Interior
Qin P.L et al. (21)	2019	China	pathology	424	484	115	133	0.93	0.98	123	2	10	113	16	Interior
Zhu J.L et al. (7)	2021	China	pathology	6760	9641	73	227	0.93	0.85	212	11	16	62	19	Interior
				6760	9641	502	530	0.95	0.90	503	50	27	452	19	Exterior
Zhou H et al. (14)	2020	China	FNA / pathology	719	448	359	224	0.84	0.88	172	72	52	287	16	Interior
				719	448	802	161	0.9	0.9	155	80	6	722	16	Exterior
Liang et al. (22)	2021	China	pathology	545	530	136	133	0.86	0.98	114	1	19	133	16	Interior
Zhu Y.C et al. (5)	2020	China	pathology	421	298	57	45	0.84	0.88	38	7	7	50	19	Interior
Zhu Y.C et al. (23)	2021	China	pathology	300	300	100	100	0.85	0.79	85	21	15	79	16	Exterior
Chan W.K et al. (6)	2021	China	pathology	4044	3316	264	204	0.81	0.8	100	14	24	56	19	Interior
Kim Y.J et al. (24)	2022	Korea	FNA	9772	2555	310	122	0.92	0.73	122	84	10	226	16	Interior
								0.87	0.68	106	99	16	211	19	Interior
				9772	2555	34	25	0.79	0.77	20	8	5	26	16	Exterior
								0.75	0.81	19	6	6	28	19	Exterior

Se, sensitivity; Sp, specificity; M, Malignant; B, Benign; TP, true positives; FP, false positives; FN, false negatives; TN, true negatives; FNA, fine needle aspiration.

### Methodology quality assessment

**Figure 2:**
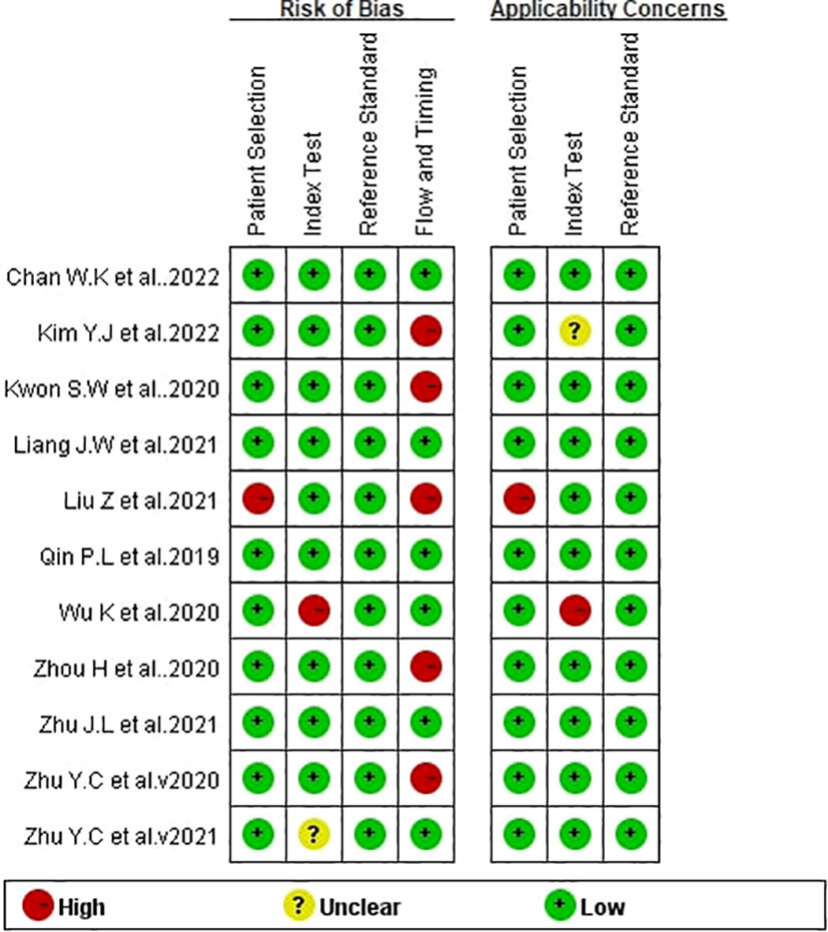
Bias risk of the included studies (QUADAS 2 criteria). The authors’ assessment of each domain for each included study.

The results of evaluating the papers’ quality assessed by the QUADAS-2 are shown in **Figure 2**. Most of the studies themselves were of high quality, but a few studies had potential risk of bias in flow and timing. In general, the included studies were considered as eligible.

### Accuracy of the ultrasound-based deep learning VGGNet model in the differential diagnosis of benign and malignant thyroid nodules

**Figure 3:**
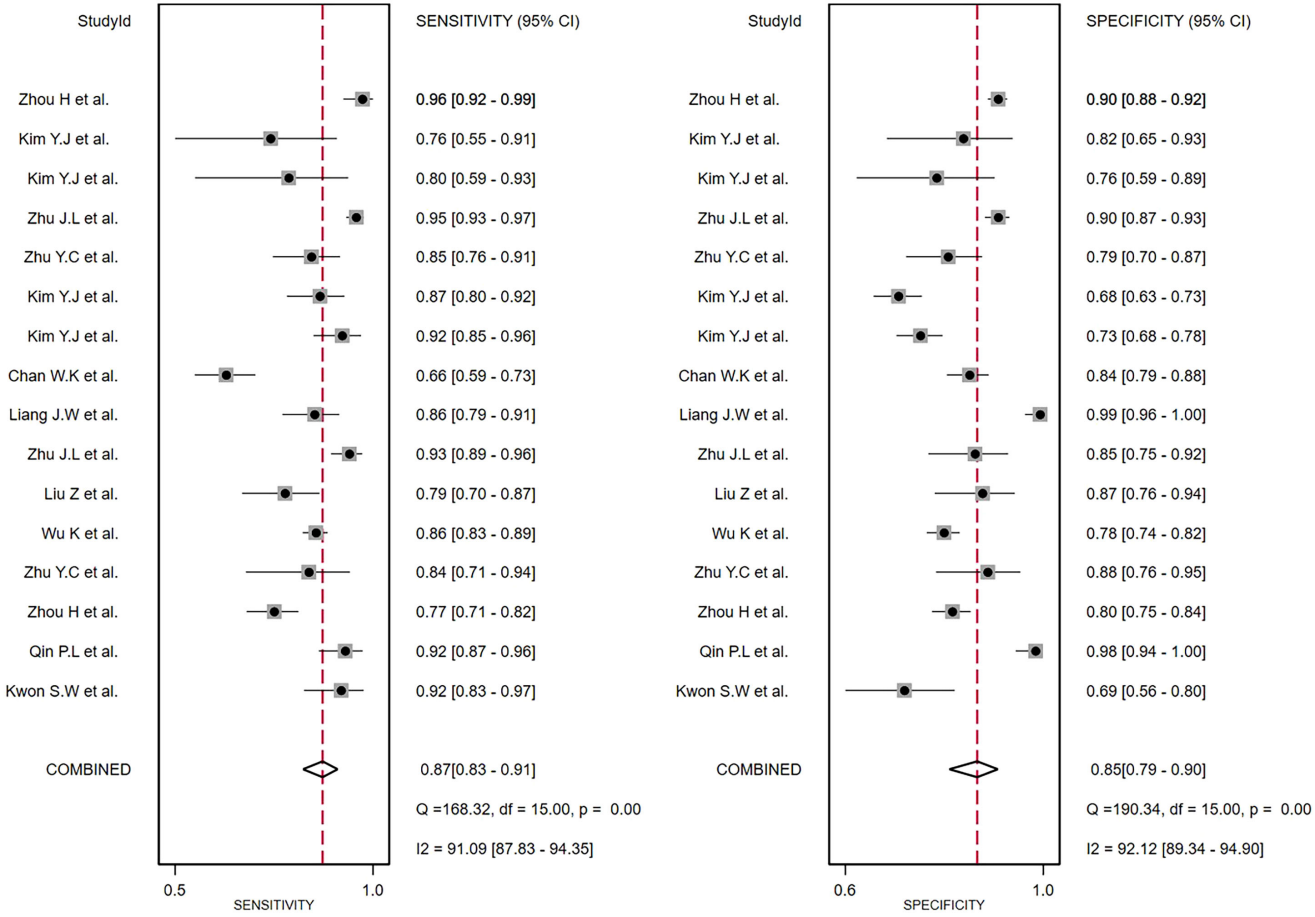
The forest plot of sensitivity and specificity for diagnosing thyroid nodules. Horizontal lines illustrate 95% confidence intervals of the individual studies.

**Figure 4:**
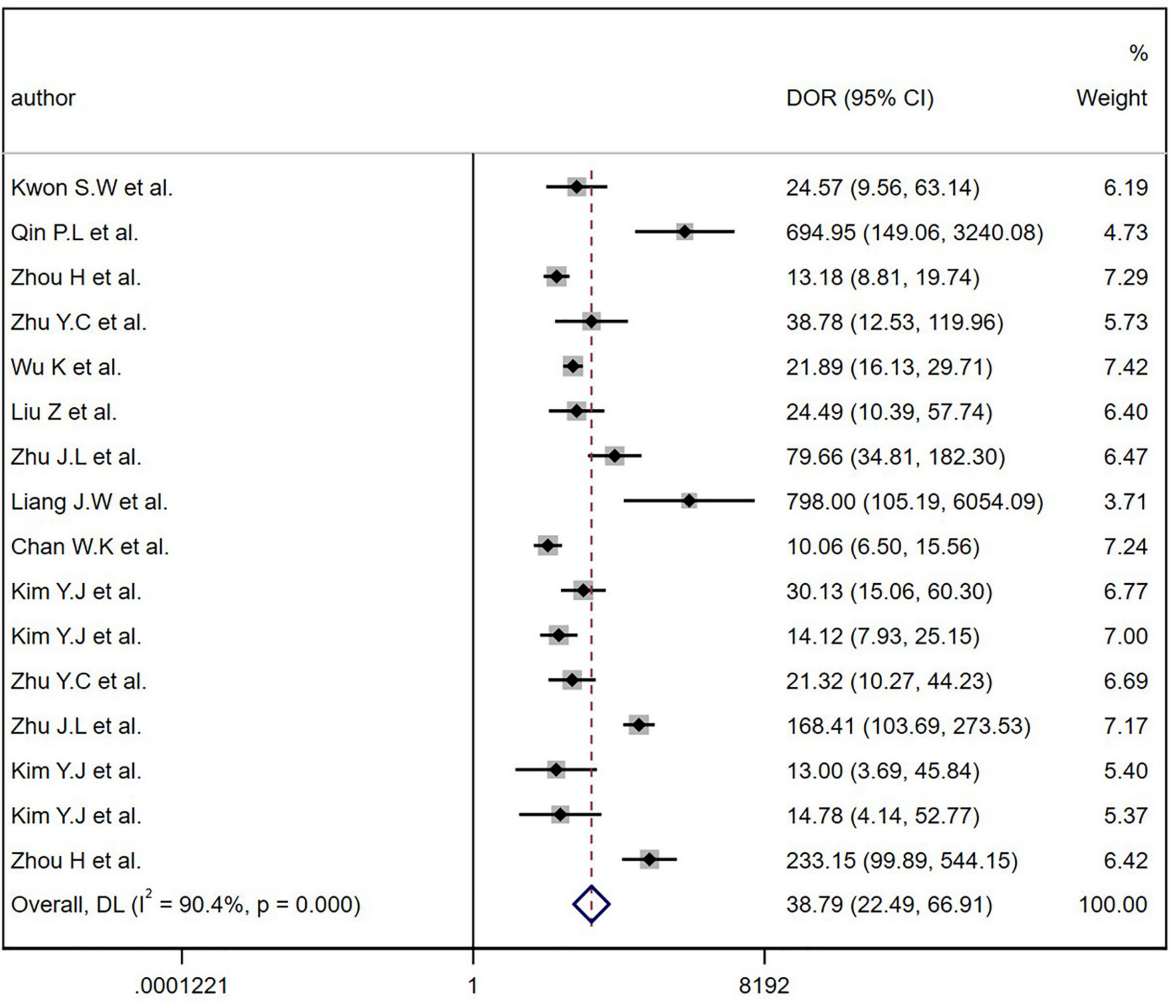
The diagnostic odds ratios (DOR) for diagnostic thyroid nodules. Horizontal lines illustrate 95% confidence intervals of the individual studies.

**Figure 5:**
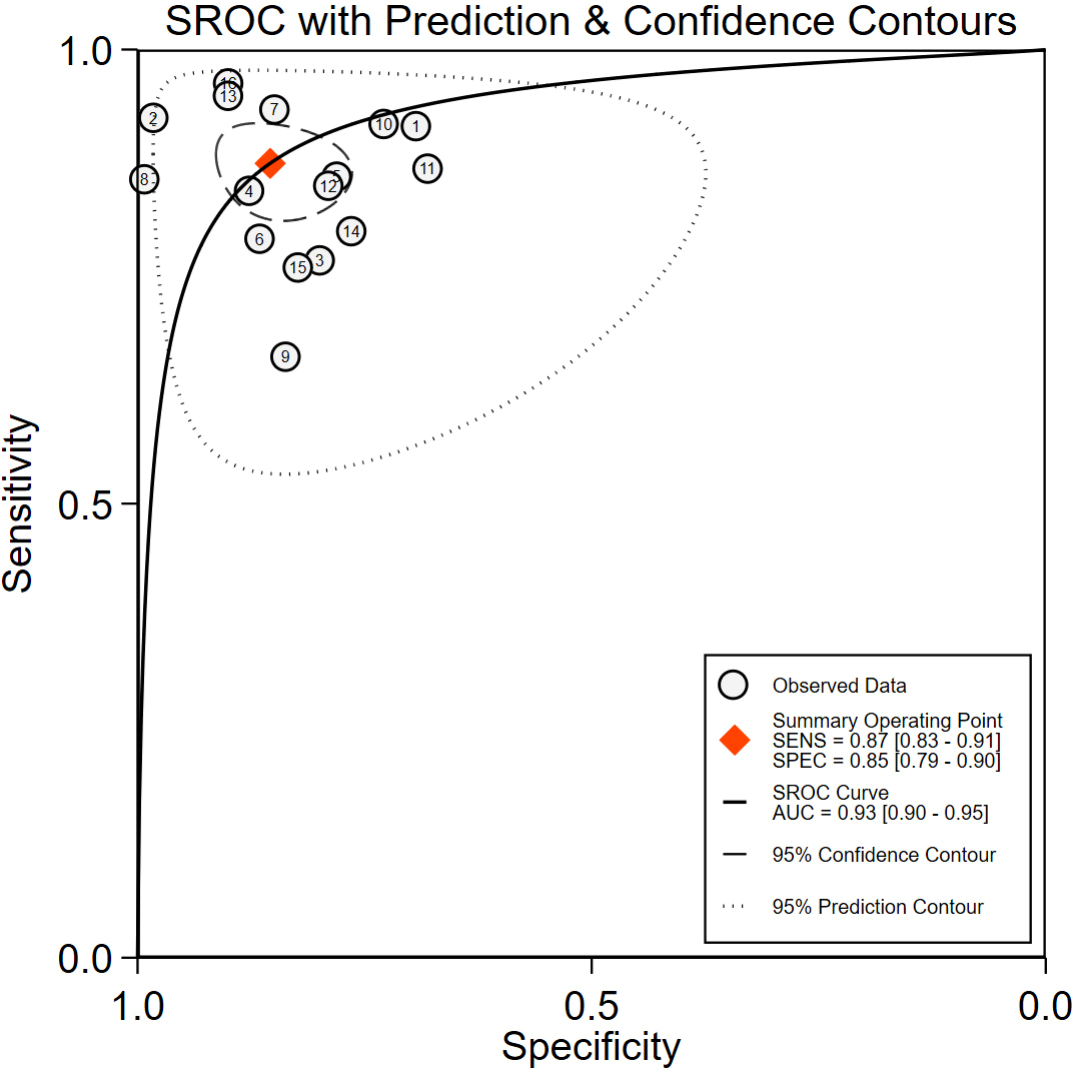
The receiver operating characteristic curve (ROC). SENS, sensitivity; SPEC, specificity; SROC, summary receiver operating characteristic curve; AUC, area under the SROC curve.

The comprehensive Pooled Sensitivity (PSEN) and Pooled Specificity (PESP) of the ultrasound-based deep learning VGGNet model for the differential diagnosis of benign and malignant thyroid nodules were 0.87 [95% CI (0.83, 0.91)] and 0.85 [95% CI (0.79, 0.90)], respectively (**Figure 3**). Higgins *I* ^2^ statistics showed significant heterogeneity in terms of sensitivity (*p* < 0.05, *I* ^2^ = 91.09%) and specificity (*p* < 0.05, *I* ^2^ = 92.12%); therefore, we selected the random-effects model to analyze the sensitivity and specificity; the DOR was 38.79 [95% CI (22.49, 66.91)] (**Figure 4**). The AUC was 0.93 [95% CI (0.90, 0.95)] (**Figure 5**). The result of Spearman correlation coefficient by Meta-DiSc version 1.4 (*r* = −0.18, *p* = 0.50) indicated that there was no significant threshold effect (*p* > 0.05), which also showed that other factors may lead to the generation of heterogeneity.

### Publication bias

**Figure 6:**
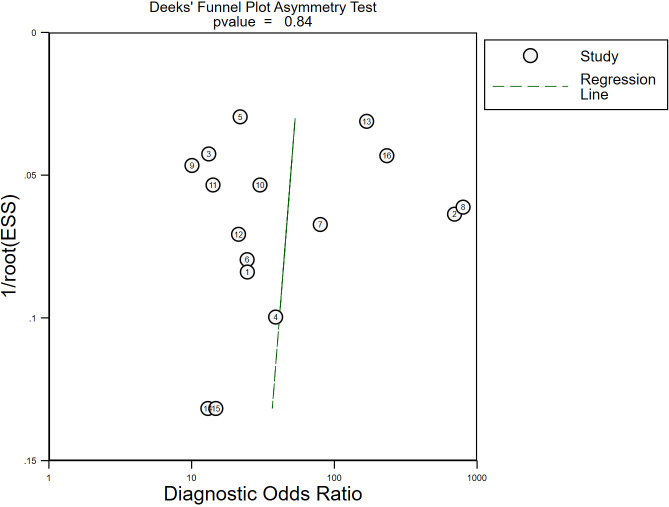
The publication bias of the included studies. No significant publication bias was found in the present meta-analysis. Each circle represented eligible research. ESS, effective sample size.

Deek’s funnel plot drawn by STATA16.0 showed no significant asymmetry, with a *p*-value of 0.84 (*p* > 0.05) (**Figure 6**), which indicated that there was no possibility of significant publication bias.

### Heterogeneity detection

Given the heterogeneity of the studies included in the pooled statistics, this research used regression analysis to analyze several clinically relevant survey variables. The result showed that year of study publication (≤2020 or >2020), number or scale of the region of interest (ROI) (single or multiple), and type of deep learning VGGNet (VGG-16 or VGG-19) were all associated with heterogeneity and were statistically significant for sensitivity (*p* < 0.05). Results of the meta-regression analysis are shown in **Table 2**. Among these covariates, the pooled sensitivity of studies published in 2020 and before was 0.89 [95% (0.84,0.95)] and 0.86 [95% (0.81, 0.91)] in papers published after 2020; the pooled specificity of papers published in 2020 and before was 0.86 [95% (0.79,0.94)] and 0.85 [95% (0.78,0.92)] in studies published after 2020, both being statistically significant (*p* < 0.05). The pooled sensitivity was 0.87 [95% (0.82, 0.91)] for a single ROI and 0.89 [95% (0.82, 0.96)] for multiple ROIs, the pooled specificity was 0.84 [95% (0.78, 0.90)] for a single ROI and 0.89 [95% (0.80, 0.97)] for multiple ROIs, and the pooled sensitivity difference was statistically significant (*p* < 0.05); the pooled specificity showed no significant differences (*p* > 0.05). The pooled sensitivity of VGG-16 was 0.88 [95% (0.83,0.93)] and VGG-19 was 0.87 [95% (0.80,0.93)], and the pooled specificity of VGG-16 was 0.86 [95% (0.80,0.93)] and VGG-19 was 0.84 [95%(0.75,0.93)], both of which were statistically significant (*p* < 0.05).

**Table 2:** Meta-regression of ultrasound-based deep learning for differentiating and diagnosing benign and malignant of thyroid nodules.

Category	*N*	Se (95% CI)	*p*	Sp (95%CI)	*p*
Year					
≤2020	6	0.89 (0.84, 0.95)	<0.05	0.86 (0.79, 0.94)	<0.05
>2020	10	0.86 (0.81, 0.91)		0.85 (0.78, 0.92)	
ROI					
Single	12	0.87 (0.82, 0.91)	<0.05	0.84 (0.78, 0.90)	0.18
Multiple	4	0.89 (0.82, 0.96)		0.89 (0.80, 0.97)	
VGG					
VGG-16	10	0.88 (0.83, 0.93)	<0.05	0.86 (0.80, 0.93)	<0.05
VGG-19	6	0.87 (0.80, 0.93)		0.84 (0.75, 0.93)	

N, number of included studies; Se, sensitivity; Sp, specificity; CI, confidence interval; ROI, region of interest.

### Sensitivity analysis

To explore whether the studies affected the stability of PSEN and PSPE, this study used a method of eliminating the literature one by one, and the results of sensitivity and specificity analysis are shown in **Table 3**. The results demonstrated that with every single paper excluded, neither PSEN and PSPE nor Higgins *I*² had significant changes. 

**Table 3:** The sensitivity analysis using the method of eliminating papers one by one.

Delete papers	Se (95% CI)	*I* ^2^ (95% CI)	*p*	Sp (95% CI)	*I* ^2^ (95% CI)	*p*	AUC (95% CI)
Zhou H et al. (14)	0.87 (0.82, 0.90)	90.54 (88.89, 94.18)	0.00	0.85 (0.78, 0.90)	91.16 (87.82, 94.49)	0.00	0.92 (0.90, 0.94)
Kim Y.J et al. (24)	0.88 (0.84, 0.91)	91.71 (88.64, 94.78)	0.00	0.86 (0.79, 0.90)	92.74 (90.15, 95.33)	0.00	0.93 (0.91, 0.95)
Kin Y.J et al. (24)	0.88 (0.83, 0.91)	91.80 (88.78, 94.83)	0.00	0.86 (0.79, 0.91)	92.75 (90.17, 95.33)	0.00	0.93 (0.91, 0.95)
Zhu J.L et al. (7)	0.87 (0.82, 0.90)	88.89 (84.40, 93.37)	0.00	0.85 (0.78, 0.90)	91.68 (88.60, 94.76)	0.00	0.92 (0.90, 0.94)
Zhu Y.C et al. (23)	0.88 (0.83, 0.91)	91.75 (88.70, 94.80)	0.00	0.86 (0.79, 0.91)	92.70 (90.09, 95.30)	0.00	0.93 (0.91, 0.95)
Kim Y.J et al. (24)	0.87 (0.83, 0.91)	91.74 (88.68, 94.79)	0.00	0.86 (0.80, 0.91)	90.04 (86.15, 93.93)	0.00	0.93 (0.91, 0.95)
Kim Y.J et al. (24)	0.87 (0.82, 0.91)	91.48 (88.29, 94.66)	0.00	0.86 (0.80, 0.91)	91.81 (88.79, 94.83)	0.00	0.93 (0.90, 0.95)
Chan W.K et al. (6)	0.89 (0.85, 0.91)	84.91 (78.27, 91.55)	0.00	0.86 (0.79, 0.90)	92.25 (89.93, 95.20)	0.00	0.93 (0.91, 0.95)
Liang J.W et al. (22)	0.88 (0.83, 0.91)	91.58 (88.45, 94.71)	0.00	0.83 (0.78, 0.87)	91.04 (87.65, 94.44)	0.00	0.92 (0.89, 0.94)
Zhu J.L et al. (7)	0.87 (0.82, 0.90)	90.89 (87.42, 94.36)	0.00	0.85 (0.79, 0.95)	92.50 (89.80, 95.19)	0.00	0.93 (0.90, 0.95)
Liu Z et al. (19)	0.88 (0.84, 0.91)	91.62 (88.51, 94.73)	0.00	0.85 (0.79, 0.90)	92.67 (90.05, 95.28)	0.00	0.93 (0.91, 0.95)
Wu K et al. (20)	0.88 (0.83, 0.91)	91.49 (88.31, 94.66)	0.00	0.86 (0.79, 0.91)	92.40 (89.66, 95.14)	0.00	0.93 (0.91, 0.95)
Zhu Y.C et al. (5)	0.88 (0.83, 0.91)	91.66 (88.57, 94.76)	0.00	0.85 (0.79, 0.90)	92.56 (89.89, 95.23)	0.00	0.93 (0.90, 0.95)
Zhou H et al. (14)	0.88 (0.84, 0.91)	90.89 (87.42, 94.36)	0.00	0.86 (0.79, 0.91)	92.52 (89.84, 95.21)	0.00	0.93 (0.91, 0.95)
Qin P.L et al. (21)	0.89 (0.84, 0.92)	91.12 (87.76, 94.47)	0.00	0.87 (0.81, 0.92)	91.35 (88.10, 94.59)	0.00	0.94 (0.92, 0.96)
Kwon S.W et al. (18)	0.87 (0.83, 0.91)	91.58 (88.45, 94.71)	0.00	0.84 (0.80, 0.91)	92.47 (89.75, 95.18)	0.00	0.93 (0.91, 0.95)

Se, sensitivity; Sp, specificity; CI, confidence interval; AUC, area under the curve.

### Fagan plot analysis

**Figure 7:**
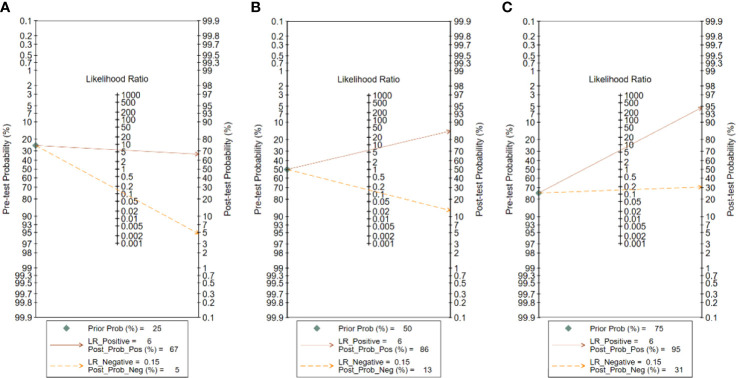
Fagan plot analysis for VGGNet model in detecting thyroid nodules: **(A)** Pre-test probability at 25%. **(B)** Pre-test probability at 50%. **(C)** Pre-test probability at 75%. The Fagan plot is composed of the left vertical axis representing the pre-test probability, the middle vertical axis representing the likelihood ratio, and the right vertical axis representing the post-test probability.

The analysis of the Fagan plots showed that the ultrasound-based deep learning VGGNet model could provide some help for radiologists on the differential diagnosis of the nature of thyroid nodules (**Figure 7**). When the prior probability was 50%, the posterior probability of the deep learning VGGNet model correctly discriminating malignant nodules as “positive” was 86%, and the posterior probability dropped to 13% when it was “negative”. When the prior probabilities were 25% and 75%, the post-test probabilities for positive were 67% and 86%, and the post-test probabilities for negative were 5% and 31%.

## Discussion

This meta-analysis evaluated the efficacy of the ultrasound-based deep learning VGGNet model in the differential diagnosis of benign and malignant thyroid nodules. The results showed that the deep learning VGGNet model achieved satisfactory results in discriminating benign and malignant thyroid nodules on ultrasound images; the pooled sensitivity and specificity were 0.87 [95% CI (0. 83, 0.91)] and 0.85 [95% CI (0.79, 0.90)], respectively, the DOR was 38.79 [95% CI (22.49, 66.91)], and the AUC was 0.93 [95% CI (0.90, 0.95)]. These results indicated that ultrasound-based deep learning VGGNet has high diagnostic accuracy for distinguishing the nature of thyroid nodules.

Traditional machine learning usually involves feature extraction and classification of ROI. Although the popularity of machine learning has gradually increased in recent years, ROI can only be manually selected and analyzed with machine learning using single-area information such as image texture, geometric shape, and statistical distribution (9). Ding et al. (25, 26) extracted statistical and textural features from thyroid elastograms, and then trained support vector machine (SVM) to detect malignancy of thyroid nodules with a maximum classification accuracy of 95.2%. However, the classification accuracy was affected by a hard threshold.

Compared with ML, deep learning can automatically extract the multi-level features of the ROI, and learn features from the nodule itself and the difference between the textures of different tissues, which greatly improves the image classification and detection performance (27). Buda et al. (28) used CNN for feature extraction and nodule classification of thyroid nodules, and also compared the diagnosis results with those of nine radiologists; the average sensitivity and average specificity of deep learning for diagnosis were higher than those of the nine radiologists, indicating that deep learning has a good clinical diagnostic value. Vasile et al. (29) used the fusion method of CNN-VGG for thyroid disease feature extraction and image classification, with an overall accuracy of 97.35%, showing that the integrative method is an excellent and stable classifier.

Previously, some meta-analyses were published about cardiovascular disease (30), gastrointestinal disease (31), and colorectal polyposis disease (32), and their combined AUCs were equal to or greater than 0.9, showing the excellent performance of CNN in disease diagnosis. Obviously, meta-analyses of thyroid nodules in ultrasound-based artificial intelligence have been conducted. Zhao et al. (33) included only five studies in meta-analysis. Xu et al. (34) mainly evaluated the overall computer-aided systems (CAD) efficacy of VGGNet, S-Detect, AlexNet, Inception, and so on in meta-analysis. In addition, the number of studies that included various single models was small, and none of them yielded the diagnostic efficacy of single-class models. Through further retrieval and reading of papers, no meta-analysis using ultrasound-based deep learning VGGNet model to differentially diagnose benign and malignant thyroid nodules has been found so far. Therefore, the authors conducted such a study.

All the included studies reported good quality, indicating that most of the included studies were of high quality and did not show significant publication bias. However, a few numbers of included studies did not inform about the continuity and randomization of case selection and the incompleteness of the implementation of the gold standard, resulting in a small number of studies with slightly poorer quality reports, which may lead to implementation bias and measurement bias, resulting in high heterogeneity. Therefore, this study chose meta-regression to explain this high degree of heterogeneity. From the results, we can see that the year of study publication, number or scale of ROI, and type of deep learning VGGNet model may be important reasons for this heterogeneity. The reasons for heterogeneity are analyzed separately in detail below.

Firstly, there were 6 sets of data from five papers published in 2020 and before (5, 14, 18, 20, 21) and 10 sets of data from six papers published after 2020 (6, 7, 19, 22–24); sensitivity and specificity were statistically significant (*p* < 0.05). The papers published after 2020 had a lower sensitivity than those published in 2020 and before (0.86 *vs*. 0.89). The reason may be that some papers published after 2020 included malignant images of thyroid nodules of different pathological types (6, 19, 23), which reduced the sensitivity of papers published after 2020. In addition, the total number of benign nodules included in papers after 2020 was less than that in 2020 and before, which reduced the specificity.

Secondly, it is easy for the ROI depicted on a single scale to ignore the rich details of ultrasound images of thyroid nodules (35). Therefore, different numbers or scales of ROIs were an important factor affecting study heterogeneity. Among the included studies, the number or scale of different studies in dividing the ROI was varied, 12 sets of data from eight papers delineated one ROI (5–7, 19–21, 23, 24), and 4 sets of data from three papers delineated two or more ROIs at different scales (14, 18, 22); sensitivity was statistically significant (*p* < 0.05). Among them, Zhou et al. (14) delineated three target regions of thyroid nodules based on average size, which were located roughly inside, around, and outside the thyroid nodule, and all three ROIs contained the nodule, which showed an AUC comparison of classification accuracy between one ROI and three ROIs (0.82 *vs*. 0.87) indicating that the classification accuracy using three ROIs was more accurate. Therefore, it is reasonable to believe that the number or scale of ROIs had an impact on the identification results of thyroid nodules.

Finally, the all included studies used the deep learning VGGNet model. The 10 sets of data from eight papers used the deep learning VGG-16 models (14, 18–24), and 6 sets of data from four papers used the deep learning VGG-19 models (5–7, 24); the paper of Kim et al. (24) had both VGG-16 and VGG-19. Our results suggested that the diagnostic sensitivity and specificity of the VGG-16 model were higher than that of the VGG-19 (0.90 *vs*. 0.79, 0.87 *vs*. 0.83); sensitivity and specificity were *p* < 0.05.* A* study had similar results, Kim et al. (24) used the VGG model to classify benign and malignant thyroid nodules on ultrasound images and compared the diagnostic accuracy of the VGG-16 model with the VGG-19 model. VGG-16 showed higher diagnostic accuracy than VGG-19 in both internal and external test sets.

Moreover, the performance of the DL model is closely connected with the number of training data, and the DL model performs better when the data of the training sample are sufficiently large (36). Based on an analysis of 11 included studies, 2 sets of data from two papers did not give an explicit number of training sets, and 14 sets of data from nine papers did give the number of training sets, but the amount of pre-training varied across studies and the amount of learning varied; thus, it is difficult to know the overfitting results of the model. In addition, some researchers have explored the use of autonomously VGGNet fine-tuned models. Currently, there is no mature deep learning CNN model that can directly differentially diagnose the nature of thyroid nodules on ultrasound, which may inevitably lead to the generation of high heterogeneity.

In addition, the Fagan plot explored the clinical utility of ultrasound-based deep learning VGGNet models. The results showed that the ultrasound-based deep learning VGGNet model had the potential to differentiate benign and malignant thyroid nodules. When a patient was considered to have a 50% chance of developing thyroid cancer after initial clinical assessment, the likelihood of developing thyroid cancer increases from 50% to 86% if the deep learning VGGNet model results appear positive. Therefore, this high probability was highly accurate. In contrast, if the deep learning VGGNet was negative, then patients had a 13% chance of thyroid cancer, which could help our radiologists to exclude thyroid cancer. In real-world clinical practice, a biopsy of masses with a predicted 25% probability of malignancy will be performed regardless of the outcome of deep learning VGGNet. Therefore, the Fagan plot showed that the deep learning VGGNet model can aid in radiologist diagnosis.

This diagnostic meta-analysis has several limitations. Firstly, studies from Europe and America were excluded because they did not meet the inclusion criteria of using the deep learning VGGNet model to differentiate benign from malignant thyroid nodules, which might cause geographic bias. Secondly, this study only included papers published in English and Chinese, which might cause an unavoidable language bias. Thirdly, this meta-analysis only included 11 papers, and the small sample size of the test set in some studies may affect the accuracy of the results of the meta-analysis. To further assess the differential diagnostic efficacy of deep learning VGGNet models, large-scale, prospective, multicenter studies in different regions are necessary.

## Conclusion

This meta-analysis suggests that the ultrasound-based deep learning VGGNet model is a suitable and effective method for radiologists to differentiate and diagnose benign and malignant thyroid nodules. However, given the limitations of the sample size and the varying quality of the studies themselves, additional prospective or multicenter studies are expected to follow for further evaluation to make up for the deficiency.

## Data availability statement

The original contributions presented in the study are included in the article/supplementary material. Further inquiries can be directed to the corresponding authors.

## Author contributions

P-SZ, JL, and X-WC contributed to the conception and design of the study. P-SZ and Y-RZ searched and reviewed studies, extracted and analyzed the data, and wrote the first draft of the manuscript. Q-LL, J-YR and X-WC reviewed and edited the manuscript. MC, TS, W-XL and JL directed the project and contributed to discussion as well as reviewed and edited the manuscript. All authors contributed to the article and approved the submitted version.

## Funding

(No. 2020-PT330-003):Supported by Open Research Fund of NHC Key Laboratory of Prevention and Treatment of Central Asia High Incidence Diseases. Supported by the Non-profit Central Research Institute Fund of Chinese Academy of Medical Sciences. (No. 2019DB012): The Corps Science and Technology Key Project.

## Conflict of interest

The authors declare that the research was conducted in the absence of any commercial or financial relationships that could be construed as a potential conflict of interest.

## Publisher’s note

All claims expressed in this article are solely those of the authors and do not necessarily represent those of their affiliated organizations, or those of the publisher, the editors and the reviewers. Any product that may be evaluated in this article, or claim that may be made by its manufacturer, is not guaranteed or endorsed by the publisher.
