# Corrigendum: Ultrasound-based deep learning using the VGGNet model for the differentiation of benign and malignant thyroid nodules: A meta-analysis

**DOI:** 10.3389/fonc.2022.1058715

**Published:** 2022-11-24

**Authors:** Pei-Shan Zhu, Yu-Rui Zhang, Jia-Yu Ren, Qiao-Li Li, Ming Chen, Tian Sang, Wen-Xiao Li, Jun Li, Xin-Wu Cui

**Affiliations:** ^1^ Department of Ultrasound, the First Affiliated Hospital of Medical College, Shihezi University, Shihezi, China; ^2^ Department of Medical Ultrasound, Tongji Hospital, Tongji Medical College, Huazhong University of Science and Technology, Wuhan, China; ^3^ NHC Key Laboratory of Prevention and Treatment of Central Asia High Incidence Diseases, First Affiliated Hospital, School of Medicine, Shihezi University, Shihezi, China

**Keywords:** meta-analysis, ultrasound, thyroid nodules, deep learning, VGGNet

In the published article, there was an error in **Table 1**. The reference numbers of each article included in this table were incorrectly marked. These citations have been changed in the table, which can be found below.

**Table 1:** Characteristics of the included studies.

Author	Year	Country	Gold standard	Training database		Test database		Se (%)	Sp (%)	TP	FP	FN	TN	VGG	Testing objects
				B	M	B	M								
Kwon S.W et al. (18)	2020	Korea	FNA/pathology	199	260	62	83	0.92	0.70	76	19	7	43	16	Interior
Liu Z et al. (19)	2021	China	FNA	–	–	67	96	0.79	0.87	76	9	20	58	16	Interior
Wu K et al. (20)	2020	China	pathology	–	–	520	636	0.86	0.78	547	114	89	406	16	Interior
Qin P.L et al. (21)	2019	China	pathology	424	484	115	133	0.93	0.98	123	2	10	113	16	Interior
Zhu J.L et al. (7)	2021	China	pathology	6760	9641	73	227	0.93	0.85	212	11	16	62	19	Interior
				6760	9641	502	530	0.95	0.90	503	50	27	452	19	Exterior
Zhou H et al. (14)	2020	China	FNA/pathology	719	448	359	224	0.84	0.88	172	72	52	287	16	Interior
				719	448	802	161	0.9	0.9	155	80	6	722	16	Exterior
Liang et al. (22)	2021	China	pathology	545	530	136	133	0.86	0.98	114	1	19	133	16	Interior
Zhu Y.C et al. (5)	2020	China	pathology	421	298	57	45	0.84	0.88	38	7	7	50	19	Interior
Zhu Y.C et al. (23)	2021	China	pathology	300	300	100	100	0.85	0.79	85	21	15	79	16	Exterior
Chan W.K et al. (6)	2021	China	pathology	4044	3316	264	204	0.81	0.8	100	14	24	56	19	Interior
Kim Y.J et al. (24)	2022	Korea	FNA	9772	2555	310	122	0.92	0.73	122	84	10	226	16	Interior
								0.87	0.68	106	99	16	211	19	Interior
				9772	2555	34	25	0.79	0.77	20	8	5	26	16	Exterior
								0.75	0.81	19	6	6	28	19	Exterior

Se, sensitivity; Sp, specificity; M, Malignant; B, Benign; TP, true positives; FP, false positives; FN, false negatives; TN, true negatives; FNA, fine needle aspiration.

In the published article, there was also an error with two reference numbers in the text. Because reference 7 used VGG-19, and reference 18 used VGG-16, the text has been modified as follows:

A correction has been made to Results, Study characteristics, Paragraph 1. The sentence “Eight papers used the deep learning VGG-16 model (7, 14, 19–23, 25)” has been corrected to “Eight papers used the deep learning VGG-16 model (14, 18–23, 25)”.

A correction has been made to Discussion, Paragraph 8. The sentence “The 11 sets of data from eight papers used the deep learning VGG-16 models (7, 14, 19–23, 25), and 6 sets of data from four papers used the deep learning VGG-19 models (5, 6, 18, 23)” has been corrected to “The 10 sets of data from eight papers used the deep learning VGG-16 models (14, 18–23, 25), and 6 sets of data from four papers used the deep learning VGG-19 models (5–7, 23)”.

In the published article, there were further errors in the text. A correction has been made to Results, Study characteristics, Paragraph 1. The sentence “Three papers did not give an explicit number of training sets (18, 19)” has been corrected to “Two papers did not give an explicit number of training sets (18, 19)”. A correction has also been made to Discussion, Paragraph 9. The sentence “2 sets of data from three papers did not give an explicit number of training sets, 14 sets of data from eight papers did give the number of training sets” has been corrected to “2 sets of data from two papers did not give an explicit number of training sets, 14 sets of data from nine papers did give the number of training sets”.

The authors apologize for these errors and state that they do not change the scientific conclusions of the article in any way. The original article has been updated.

## Publisher’s note

All claims expressed in this article are solely those of the authors and do not necessarily represent those of their affiliated organizations, or those of the publisher, the editors and the reviewers. Any product that may be evaluated in this article, or claim that may be made by its manufacturer, is not guaranteed or endorsed by the publisher.

